# Procognitive Effects of Adjunctive D-Cycloserine to Intermittent Theta-Burst Stimulation in Major Depressive Disorder: Effets procognitifs de la D-cyclosérine en traitement complémentaire par la stimulation thêta-burst intermittente dans le trouble dépressif caractérisé

**DOI:** 10.1177/07067437241293984

**Published:** 2024-10-29

**Authors:** Marilena M. DeMayo, Jaeden Cole, Myren N. Sohn, Signe L. Bray, Ashley D. Harris, Scott B. Patten, Alexander McGirr

**Affiliations:** 1 Department of Psychiatry, 2129 University of Calgary, Calgary, Canada; 2 157742 Hotchkiss Brain Institute, 2129 University of Calgary, Calgary, Canada; 3 550962 Mathison Centre for Mental Health Research and Education, Calgary, Canada; 4 Department of Radiology, 2129 University of Calgary, Calgary, Canada; 5 Alberta Children's Hospital Research Institute, 2129 University of Calgary, Calgary, Canada; 6 Department of Community Health Sciences, 2129 University of Calgary, Calgary, Canada

**Keywords:** cognition, working memory, D-cycloserine, NMDA receptor, repetitive transcranial magnetic stimulation, rTMS, intermittent theta burst stimulation, iTBS, major depressive disorder, MDD., cognition, mémoire de travail, D-cyclosérine, récepteur NMDA, stimulation magnétique transcrânienne répétitive, rTMS, intermittente stimulation thêta burst, iTBS, trouble dépressif majeur, TDM

## Abstract

**Objective:**

Major depressive disorder (MDD) is associated with cognitive impairments that persist despite successful treatment. Transcranial magnetic stimulation is a noninvasive treatment for MDD that is associated with small procognitive effects on working memory and executive function. We hypothesized that pairing stimulation with N-methyl-D-aspartate (NMDA) receptor agonism would enhance the effects of stimulation and its procognitive effects.

**Method:**

The effect of NMDA receptor agonism (D-cycloserine, 100 mg) on cognitive performance was tested in two randomized double-blind placebo-controlled trials: (1) acute effects of in the absence of stimulation (*n *= 20 healthy participants) and (2) a treatment study of individuals with MDD (*n *= 50) randomized to daily intermittent theta-burst stimulation (iTBS) with placebo or D-cycloserine for 2 weeks. Cognitive function was measured using the THINC-it battery, comprised of the Perceived Deficits Questionnaire, the Choice Reaction Time, the Trail Making Test, the Digit Symbol Substitution Test, and the 1-Back tests.

**Results:**

D-cycloserine had no acute effect on cognition compared to placebo. iTBS + D-cycloserine was associated with significant improvements in subjective cognitive function and correct responses on the 1-Back when compared to iTBS + placebo. Improvements in subjective cognition paralleled depressive symptoms improvement, however 1-Back improvements were not attributable to improvement in depression.

**Conclusions:**

An intersectional strategy pairing iTBS with NMDA receptor agonism may restore cognitive function in MDD.

## Résumé

**Objectif:**

Le trouble dépressif caractérisé (TDC) est associé à des déficits cognitifs qui persistent malgré la réussite du traitement. La stimulation magnétique transcrânienne est un traitement non invasif du TDC qui est associé à de faibles effets procognitifs sur la mémoire à court terme et la fonction exécutive. Nous avons émis l’hypothèse que l’association de la stimulation et de l’agonisme des récepteurs NMDA renforcerait les effets de la stimulation et ses effets positifs sur les performances cognitives.

**Méthode:**

L’effet de l’agonisme des récepteurs NMDA (D-cyclosérine, 100 mg) sur les performances cognitives a été testé dans deux essais randomisés à double insu et contrôlés par placebo: (1) les effets aigus en l’absence de stimulation (*n *= 20 participants sains); (2) une étude sur le traitement des personnes atteintes de TDC (*n *= 50) réparties aléatoirement pour recevoir une stimulation thêta-burst intermittente (STBi) associée au placebo ou à la D-cyclosérine pendant deux semaines. La fonction cognitive a été mesurée à l’aide de l’outil THINC-it, composé du Perceived Deficits Questionnaire, de la mesure du temps de réaction de choix, du Trail Making Test, du Digit Symbol Substitution Test et du One-Back Test.

**Résultats:**

La D-cyclosérine n’a eu aucun effet aigu sur la cognition par rapport au placebo. L’association STBi-D-cyclosérine a été associée à des améliorations significatives de la fonction cognitive subjective et à des bonnes réponses au One-Back Test par rapport à l’association STBi-placebo. Les améliorations sur le plan de la cognition subjective étaient parallèles à l’amélioration des symptômes dépressifs, mais la meilleure performance au One-Back Test n’était pas attribuable à l’amélioration des symptômes dépressifs.

**Conclusion:**

Il se peut qu’une stratégie intersectionnelle associant la STBi à l’agonisme des récepteurs NMDA restaure la fonction cognitive chez les personnes souffrant de TDC.

## Introduction

Cognitive difficulties are a core symptom of major depressive disorder (MDD)^1^ and range from attention, response inhibition, learning and memory, and executive function.^2–5^ They are associated with poor psychosocial functioning^6^ and appear to persist despite successful treatment.^3,4^

Means of restoring cognitive function is an important and unmet need in the treatment of MDD, with potential relevance to other conditions impacting cognitive function. Noninvasive neurostimulation may provide a unique opportunity to restore cognition by selectively targeting neural circuits implicated in dysfunction. Notably, repetitive transcranial magnetic stimulation (rTMS), the modality with the largest evidence base in MDD,^7,8^ is associated with small but statistically significant improvements in working memory (Hedges’ *g *= –0.198) and cognitive control/executive function (Hedges’ *g *= –0.23).^9^

The presumed mechanism of action of rTMS and intermittent theta-burst TMS (iTBS) involves synaptic plasticity and long-term potentiation (LTP)-like or long-term depression (LTD)-like effects that result from stimulation. We and others have demonstrated that N-methyl-D-aspartate (NMDA) receptor agonism with the pharmacological adjunct, D-cycloserine, enhances LTP-like and LTD-like effects of rTMS^10^ and iTBS^11^ in the motor cortex, and significantly enhances antidepressant efficacy when repeatedly administered as a treatment for MDD,^12^ aligning with the stated antidepressant effects of NMDA receptor modulation.^13^ We hypothesized that pairing stimulation with NMDA receptor agonism would enhance the procognitive effects of iTBS, and be selective to the cognitive domains that are subserved by the dorsolateral prefrontal cortex (DLPFC) such as working memory. Further, given that previous data examining procognitive effects of D-cycloserine have been mixed, we explored the effects of D-cycloserine on our battery in a convenience sample of healthy individuals participating in a randomized controlled crossover trial.

## Methods and Materials

### Included Trials

Participants from two pre-registered randomized placebo-controlled double-blind trials are included (NCT05081986 and NCT03937596). These studies were both conducted with approval from the Conjoint Health Research Ethics Board of the University of Calgary, and written informed consent was obtained. Data was collected at the University of Calgary. The primary outcomes from these studies have been published elsewhere,^11,12^ however, both studies included cognition as a secondary outcome.

The first trial (NCT05081986) involved a randomized double-blind placebo-controlled crossover design testing the acute effects of D-cycloserine (100 mg) or placebo in a convenience sample of *n *= 20 healthy individuals^11^ participating in a motor plasticity study. Single doses were administered on two occasions, counterbalanced placebo/D-cycloserine, with a crossover condition 1 week later.

The second trial (NCT03937596) involved a randomized double-blind placebo-controlled design with *n *= 50 individuals with MDD who received daily D-cycloserine (100 mg) or placebo as an adjuvant to iTBS for 2 weeks, with a 1:1 ratio.^12^ Participants received blister packs with their randomized medication, and were instructed to take one capsule daily on weekdays prior to their iTBS session. Inclusion criteria were a primary diagnosis of MDD by Diagnostic and Statistical Manual of Mental Disorders, 5th edition (DSM-5) criteria, experiencing a major depressive episode with score ≥18 on the 17-item Hamilton Depression Rating Scale (HDRS-17), failure to achieve clinical response with at least one adequate trial of a first-line antidepressant medication or psychotherapy but not more than 4 adequate trials in the current episode, stable medications for 4 weeks prior to enrolment and throughout the study, as well as normal blood work including a complete blood count, electrolytes, liver function tests, and creatinine. Exclusion criteria included a history of nonresponse to rTMS or electroconvulsive therapy (ECT), and the initiation of psychotherapy within 3 months of enrollment or during the trial.

### Cognitive Measurements

Participants completed the THINC-it, a computerized cognitive battery developed as a cognitive screening tool focused on domains impacted in MDD.^14^ The THINC-it has been validated against established cognitive tests,^15^ demonstrated to be sensitive to the cognitive deficits seen in MDD,^14^ and to have test–retest reliability.^15,16^ It includes five components that are validated variations of established cognitive tests:

1. The “Spotter” based on the Choice Reaction Time (CRT) involves 40 trials in which subjects are presented with an arrow facing either left or right. Participants are asked to press a button corresponding to the direction of the arrow. Latency to response is recorded, and shorter reaction time indicates better performance and attention.
2. The “Symbol Check” is based on the 1-Back test. It involves 40 trials in which a continuously moving sequence of symbols moves across the screen. As the sequence moves to the left, the participant is asked to identify the previous symbol, now hidden, before time-out (3 s). A legend of five possible symbols is presented at the bottom of the screen. Accuracy in the form of number correct is recorded, with higher number of correctly identified symbols indicating better performance and working memory.
3. The “Trails” is based on Part B of the Trail Making Test (TMT). Participants are asked to trace a line connecting consecutive numbers and letters in alternation. If the line touches a number or letter out of sequence, the participant must restart beginning at “A.” Completion time is recorded, with lower times indicating better performance and executive function.
4. The “Codebreaker” is based on the Digital Symbol Substitution Test (DSST). It is a 2-min test in which participants are presented with a series of numbers and are asked to match numbers with its associated symbol. The corresponding legend of six symbols is located at the top of the screen. The number of correct responses is recorded, with more correct symbols indicative of better performance and cognitive function across several domains including working memory, attention, and executive function.
5. The “Perceived Difficulties Questionnaire” (PDQ) is a five-item questionnaire assessing perceived challenges with attention, memory, and concentration in the past 7 days. Each item is rated on a Likert scale ranging from 1 (“Never”) to 5 (“Very Often [More than once a day]”). The total score is recorded, and higher scores are indicative of greater subjective cognitive impairment.

Healthy participants completed the THINC-it approximately 2 h after ingesting either placebo or D-cycloserine in a counterbalanced crossover design separated by at least 7 days. Participants with MDD completed the THINC-it prior to initiating iTBS treatments and after 2 weeks of iTBS treatment paired with the D-cycloserine or placebo.

### Study Drug

D-cycloserine was purchased as Seromycin 250 mg capsules (Parsolex GCMP Center, West Lafayette, IN) and repackaged as capsules containing 100 mg of D-cycloserine. Placebo capsules contained 100 mg microcrystalline cellulose. The capsules were visually identical and instructions regarding dosing were the same for both the placebo and D-cycloserine capsules.

### Randomization

A random number sequence was used to generate a 1:1 randomization sequence for each respective study. Eligible participants were randomized with allocation concealment. Eligible participants, the research team, and outcome assessors remained blind to treatment condition throughout the duration of the study.

### Intermittent Theta Burst Stimulation

In the trial investigating the combination of iTBS and D-cycloserine for MDD, we utilized a MagPro X100 stimulator (MagVenture, Denmark) and a COOL-B70 coil. Targeting of the left DLPFC involved surface anatomy using the Beam F3 method.^17^ Resting motor threshold (rMT) was determined using electromyographic (EMG) electrodes placed over the first dorsal interosseous muscle, with threshold determined as the minimum stimulus intensity required to elicit at least 5 out of 10 EMG responses of ≥50 µV. iTBS consisted of a total of 600 pulses per session delivered in 20 trains of triplets at 50 Hz repeated at 5 Hz (2 s on 8 s off) at 80% rMT. Participants received daily treatments Monday through Friday for a total of 20 treatments. For the first 2 weeks, participants took their adjunctive medication (D-cycloserine or placebo) prior to iTBS treatment, for a total of 10 treatments.

### Mood Assessments

Participants with MDD were assessed by a psychiatrist blind to treatment condition using the clinician rated Montgomery-Åsberg Depression Rating Scale (MADRS).^18^

### Power Calculation

Power calculations were not conducted based on subdomains of the THINC-it but rather based on the primary outcomes of each study: corticospinal excitability for the healthy convenience sample and depressive symptoms for the MDD treatment trial. Both studies had 95% power with alpha of <0.05 to detect a small effect (acute dose Cohen's f: 0.3; MDD treatment trial Cohen's f: 0.2).

### Statistical Analyses

Unblinded analyses were performed using SPSS (v28). Outliers were identified using interquartile range, with threshold set at values more than three interquartiles. Alpha was set at 0.05 with Bonferroni adjusted alpha of 0.01 for 5 THINC-it domains. Comparisons between healthy participants and participants with MDD utilized independent group *t*-tests and one-way analysis of variance (ANOVA). To investigate the effect of the interventions, intention-to-treat (ITT) analyses were used, with repeated measures ANOVA with last observation carried forward as this is a conservative method of imputing missing data. To better account for missing data, analyses were repeated as generalized linear mixed effect models. In the MDD sample, additional analyses covarying for percent change in MADRS score were conducted.

## Results

### Acute Effects of D-Cycloserine on Cognition

Twenty healthy participants (7 females/13 males; 33.35 ± 9.57 years old) participated in the acute dosing crossover study. Participants reported Asian (*n *= 2), African (*n *= 1), European (*n *= 16), and Latin Central and South American (*n *= 1) origins. Two participants are missing data due to computer problems.

**Figure 1.**
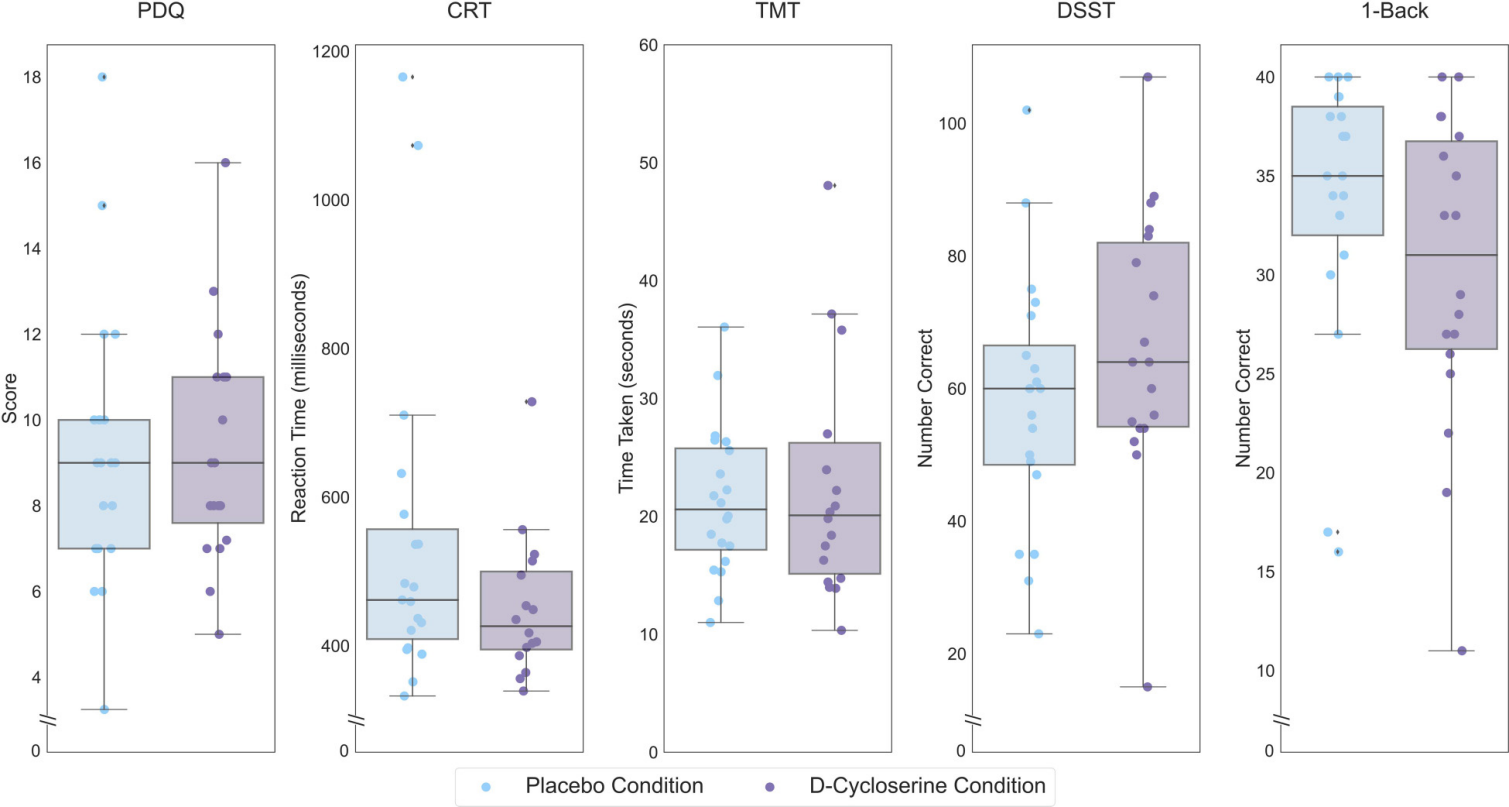
Performance of healthy controls on each THINC-it subtest following placebo administration, lighter hue, and D-cycloserine administration, darker hue. Scores are performance on the THINC-it domains (PDQ, CRT, TMT, DSST, and 1-Back). Lower scores indicate less subjective cognitive difficulties for the PDQ, lower values indicate better performance for the CRT and TMT while higher values indicate better performance for the DSST and 1-Back. Boxes represent the interquartile range, with the median represented by the center line, while whiskers are 1.5 the interquartile range. CRT: Choice Reaction Time; DSST: Digit Symbol Substitution Test; PDQ: Perceived Deficits Questionnaire; TMT: Trail Making Test.

There was a significant effect of D-cycloserine on performance on the DSST (*F*(1,18) = 4.65, *p *= 0.045, partial eta-squared = 0.205; Figure 1), that did not survive correction for multiple comparisons. No other subtests showed a significant effect of acute D-cycloserine on cognitive performance.

### THINC-it Cognitive Domains in Participants With MDD

**Figure 2.**
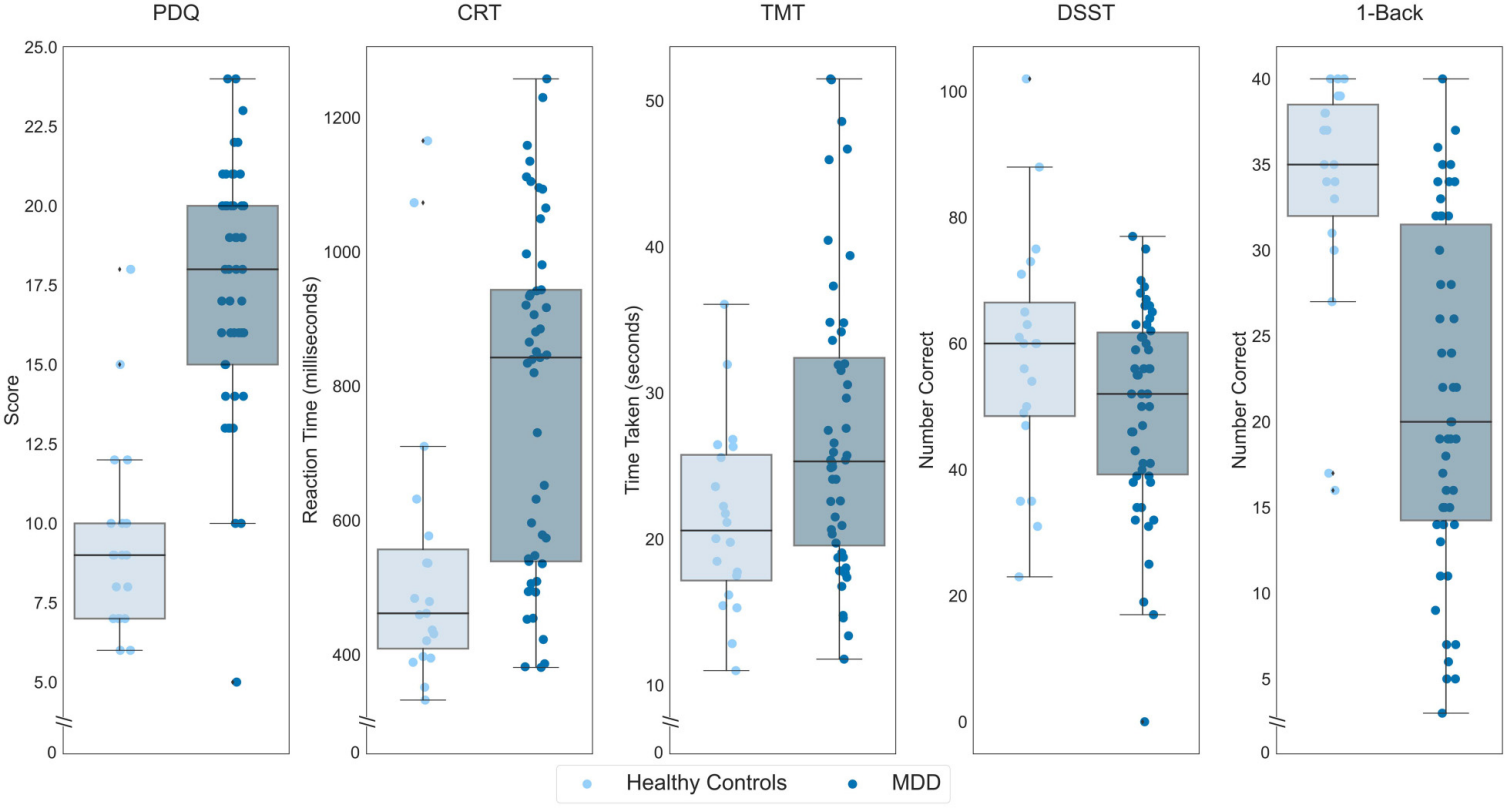
Performance of participants with MDD, in darker hue, and healthy controls in the placebo condition, lighter hue, on each THINC-it subtest. On the y-axis, lower scores indicate less subjective cognitive difficulties for the PDQ, lower values indicate better performance for the CRT and TMT while higher values indicate better performance for the DSST and 1-Back. Boxes represent the interquartile range, with the median represented by the center line, while whiskers are 1.5 the interquartile range. CRT: Choice Reaction Time; DSST: Digit Symbol Substitution Test; MDD: major depressive disorder; PDQ: Perceived Deficits Questionnaire; TMT: Trail Making Test.

We first sought to confirm the sensitivity of the THINC-it to cognitive impairments in MDD, for which it was designed, by comparing our healthy individuals (*n *= 20) with the baseline performance of *n *= 50 individuals with MDD who participated in the treatment trial (Figure 2). These comparisons revealed greater perceived deficits among participants with MDD on the PDQ (*t*(68) = 8.28, *p *< 0.001), slower reaction time on the CRT (*t*(37.10) = 3.82, *p *< 0.001), greater time required to complete the TMT (*t*(55.79) = 2.94, *p *= 0.002) and fewer correct responses on the 1-Back (*t*(45.76)=−5.74, *p *< 0.001). There was not a significant difference between the two groups on the DSST (*t*(68)=−1.81, *p *= 0.074). Repeating these analyses accounting for age, gender, and years of education, there was a significant difference between participants with MDD and healthy controls on the PDQ (*F*(1,64) = 54.24, *p *< 0.001) and the 1-Back (*F*(1,64) = 4.21, *p *= 0.044), while the differences between groups on the CRT (*F*(1,63) = 2.193, *p *= 0.14), TMT (*F*(1,64) = 0.001, *p *= 0.97), and DSST (*F*(1,64) = 0.97, *p *= 0.33) were not significant.

### Changes in Cognition With iTBS and Placebo-Controlled Adjunctive NMDA Receptor Agonism in MDD

Fifty individuals with MDD were randomized to receive iTBS + placebo (*n *= 25; 15 females/10 males; 40.88 ± 13.36 years old, 5 not on psychiatric medication) or iTBS + D-cycloserine (*n *= 25; 16 females/9 males; 40.64 ± 13.64 years old, 3 not on psychiatric medication). Participants reported Asian (*n *= 7), European (*n *= 37), Latin, Central and South American (*n *= 3), Middle Eastern (*n = *1), and Mixed (*n *= 2) origins. The iTBS + placebo and iTBS + D-cycloserine groups did not statistically differ on sociodemographic or clinical characteristics including depressive symptom severity, MADRS score or concomitant treatments.^12^

#### ITT Analysis

**Figure 3.**
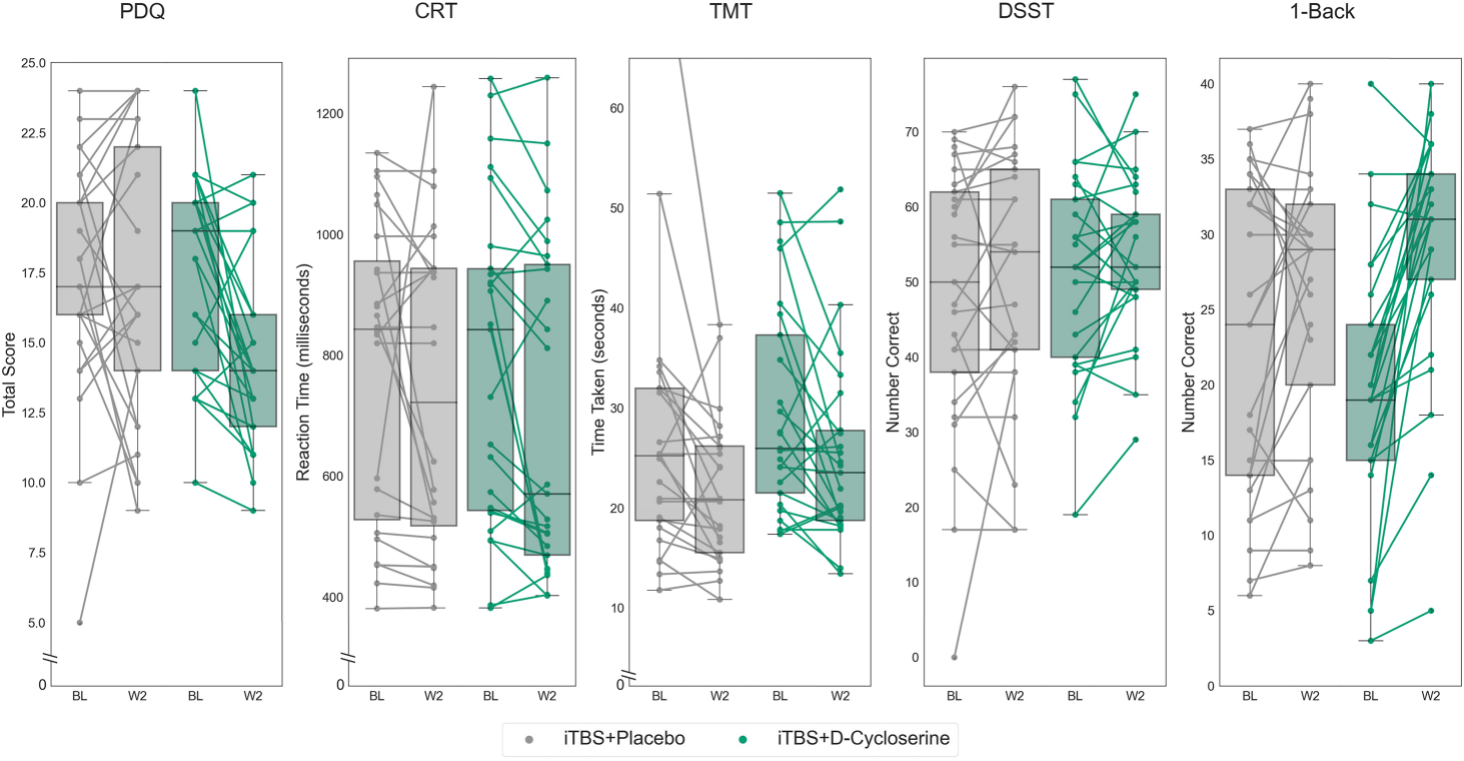
THINC-it subtest scores at baseline and after 2 weeks of iTBS stimulation paired with either placebo (iTBS + placebo) in lighter hue or with adjunctive D-cycloserine (iTBS + D-cycloserine) in darker hue. Data shown is all randomized participants (intention-to-treat sample). Boxes represent the interquartile range, with the median represented by the center line, while whiskers are 1.5 the interquartile range. BL: baseline; iTBS: intermittent theta-burst stimulation; W2: week 2.

The effects of iTBS + placebo and iTBS + D-cycloserine are illustrated in Figure 3. There was a significant GROUP*TIME interaction (iTBS + placebo or iTBS + D-cycloserine) for the 1-Back (*F*(1,47) = 10.21, *p *= 0.002, partial eta-squared = 0.178) and for the PDQ (*F*(1,48) = 5.12, *p *= 0.028, partial eta-squared = 0.135), with iTBS + D-cycloserine participants showing greater improvements than iTBS + placebo participants on both domains, however only the 1-Back remained statistically significant following correction for multiple comparisons. There were no significant GROUP*TIME interaction effects on the CRT (*F*(1,47) = 0.57, *p *= 0.46), DSST (*F*(1,48) = 0.02, *p *= 0.89), or TMT (*F*(1,48) = 1.25, *p *= 0.27).

We repeated analyses relating to the PDQ and the 1-Back to determine whether these effects were attributable to antidepressant superiority of the iTBS + D-cycloserine treatment condition. Controlling for percent change on the MADRS from baseline, the 1-Back continued to show a significant GROUP*TIME interaction (*F*(1,46) = 8.54, *p *= 0.005, partial eta-squared = 0.157), with no significant interaction between MADRS_change*TIME (*F*(1,46) = 2.74, *p *= 0.077). For the PDQ, this interaction was no longer significant after controlling for improvements in depressive symptoms (GROUP*TIME (*F*(1,47) = 3.15, *p *= 0.082).

#### Mixed Model Analysis

The analysis was repeated as linear mixed model for the 1-Back and PDQ. For the 1-Back there was an effect of TIME (*F*(1,94) = 13.66, *p* < 0.001) and a trend effect of GROUP*TIME interaction for the 1-Back (*F*(1,94) = 3.37, *p *= 0.069), with no main effect of GROUP (*F*(1,94) = 0.16, *p *= 0.69). For the PDQ, there was a significant effect of TIME (*F*(1,96) = 6.47, *p* = 0.013), and no effect of GROUP (*F(*1,96) = 0.991, *p *= 0.32) or GROUP*TIME interaction (*F(*1,96) = 1.31, *p *= 0.26).

Repeating these analyses while controlling for improvement on the MADRS revealed a significant effect of TIME (*F*(1,59.74) = 23.84, *p *< 0.001) and a significant interaction between GROUP*TIME (*F*(1,59.74), *p *= 0.006) for the 1-Back with no significant GROUP (*F*(1,58.32) = 0.002, *p *= 0.96) effect. For the PDQ, there was a significant effect of TIME *F*(1,55.53) = 8.43, *p *= 0.005), with no effect of GROUP (*F*(1,58.70) = 0.58, *p *= 0.45) or GROUP*TIME (*F*(1,55.53) = 1.94, *p *= 0.17) interaction.

#### Post-Treatment Comparisons to Healthy Controls

**Figure 4.**
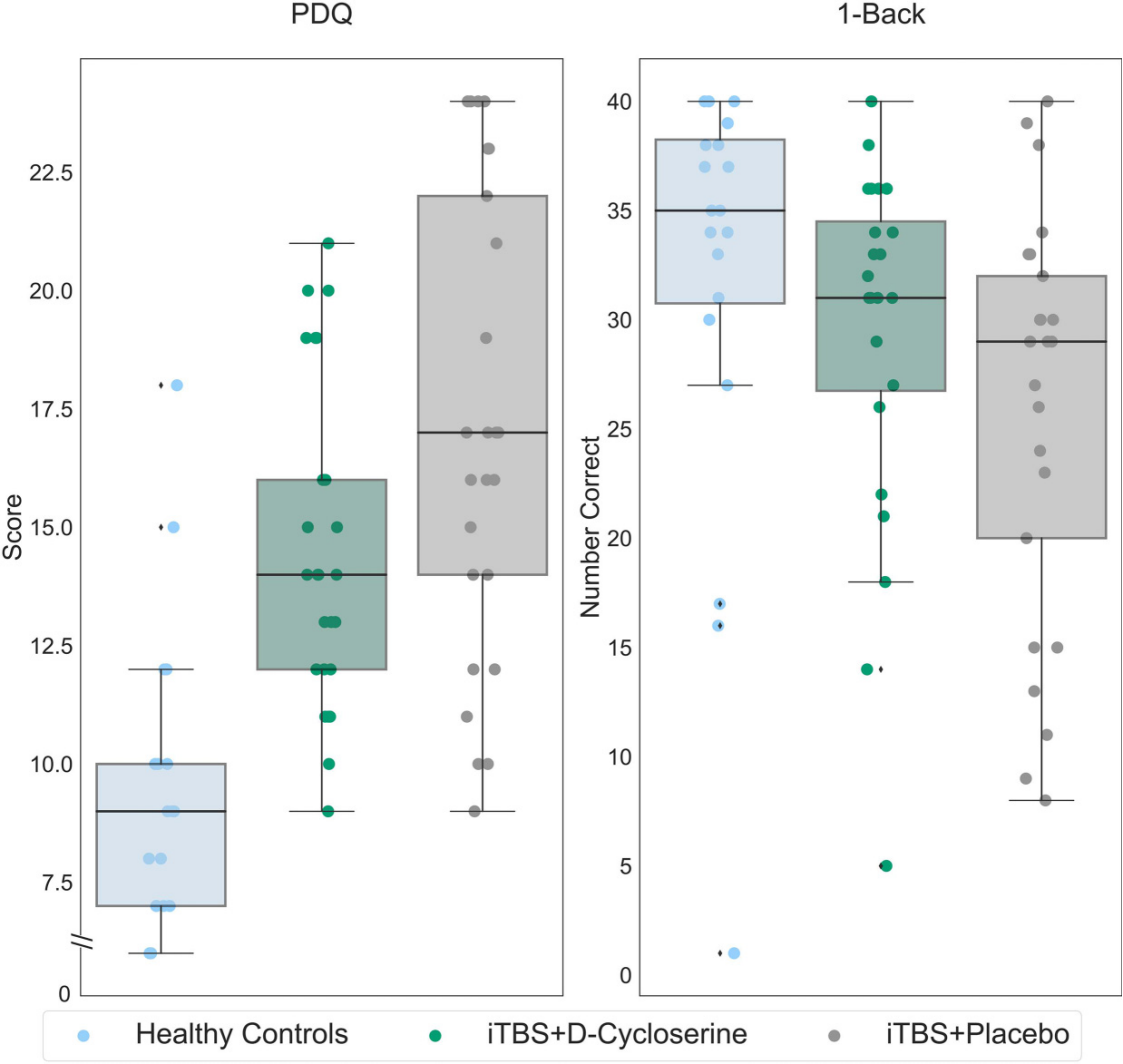
1-Back and PDQ scores for healthy controls (light blue), participants with MDD who received iTBS + D-cycloserine (dark green) and participants with MDD who received iTBS + placebo (gray). Data shown is all randomized participants (intention-to-treat sample). On the y-axis, lower scores indicate less subjective cognitive difficulties for the PDQ, while higher values indicate better performance for the 1-Back. Boxes represent the interquartile range, with the median represented by the center line, while whiskers are 1.5 the interquartile range. iTBS: intermittent theta-burst stimulation; MDD: major depressive disorder; PDQ: Perceived Deficits Questionnaire.

Post-treatment scores for the iTBS + D-cycloserine and iTBS + placebo groups were compared to the healthy controls (Figure 4). After 2 weeks of treatment, a one-way ANOVA showed no significant differences between the iTBS + D-cycloserine-treated participants, iTBS + placebo treated participants and healthy controls on the 1-Back (*F*(2,68) = 2.59, *p *= 0.082). Post hoc comparisons showed a significant difference between iTBS + placebo treated participants and healthy controls (*p *= 0.027, 95% confidence interval (CI) = 11.70, 0.72) but not iTBS + D-cycloserine-treated participants and healthy controls (*p *= 0.32, 95% CI = −2.79, 8.30). Significant group differences remained for the PDQ (*F*(2,69) = 21.21, *p *< 0.001), with both participants who received iTBS + D-cycloserine (*p *< 0.001, 95% CI = −7.43, −2.71) and those who received iTBS + placebo (*p *< 0.001, 95% CI = −9.98, −5.27) continued to report more cognitive deficits on the PDQ than healthy controls.

Repeating these analyses accounting for gender, age, and education, a similar pattern of results held. For the 1-Back, there was no significant differences between the iTBS + D-cycloserine treated participants, iTBS + placebo treated participants and healthy controls (*F*(2,61) = 0.53, *p *= 0.59). Post hoc comparisons showed no difference between iTBS + placebo treated participants and healthy controls (*p *= 0.59, 95% CI=−9.35, 3.74) and iTBS + D-cycloserine treated participants and healthy controls (*p *= 0.89, 95% CI=–6.94, 6.02). Significant group differences remained with these covariates for the PDQ (*F*(2,62) = 18.31, *p *< 0.001), with both participants who received iTBS + D-cycloserine (*p *< 0.001, 95% CI = 2.93, 8.67) and those who received iTBS + placebo (*p *< 0.001, 95% CI = 5.82,11.56) reporting more cognitive deficits on the PDQ than healthy controls.

## Discussion

Our data suggests that pairing iTBS with an adjunct targeting the NMDA receptor significantly improves objective and subjective cognitive function in MDD. Moreover, our data suggests that pharmacologically enhanced iTBS may restore working memory to the level of healthy controls and population norms.^15^ It is important to note that working memory has previously been shown to improve with TMS in recent meta-analyses, however with a much smaller effect,^9^ thereby supporting the interpretation that pharmacological enhancement of iTBS synaptic plasticity may also increase procognitive effects. Conversely, significant improvements in subjective cognitive function paralleled improvements in depressive symptoms, suggesting that subjective gestalt cognitive function tracks syndromic severity of depressive symptoms.

Our findings regarding working memory are consistent with the a priori hypothesis that a mechanistically informed adjunct would selectively enhance iTBS plasticity within the DLPFC and its associated circuitry. This was hypothesized because the DLPFC is critical to working memory,^19^ with the acknowledged limitation that the 1-Back task is not as heavily reliant on the DLPFC as an N-Back task.^20^ Moreover, improvements in working memory effect could not be accounted for by improvements in clinician rated depressive symptoms. We speculatively propose that pairing iTBS with D-cycloserine resulted in functional remodeling in the DLPFC and associated circuitry. This interpretation is consistent with the NMDA-receptor dependent nature of iTBS^21^ and our previous data in motor cortex demonstrating the normalization and stabilization of synaptic plasticity in individuals with MDD with this mechanistically informed adjuvant.^22^

Our analyses suggest that, in healthy individuals, an acute administration of D-cycloserine is not associated with cognitive enhancement in THINC-it domains, however, this conclusion is tempered by the small sample. This is consistent with previous studies examining related tests in healthy individuals^23^ and individuals with schizophrenia.^24^ Nevertheless, there is data to support cognitive enhancement with the dose of D-cycloserine utilized in this sample in a dementia sample,^25^ suggesting that the effects of NMDA receptor agonism and cognition may be population specific. The broader literature examining D-cycloserine and cognitive enhancement in healthy individuals is mixed, with some data supporting greater enhanced fear learning,^26,27^ procedural learning,^28^ declarative learning,^29^ probabilistic learning,^30^ and visuospatial reasoning,^31^ while other data suggests the absence of an effect on fear learning^32^ or using a tactile discrimination task.^33^ Dosing in these studies ranged from 50 to 500 mg, thereby spanning serum concentrations sufficient to agonize and antagonize the NMDA receptor, however, this does not account for the discrepant findings. Additional study on the effects of D-cycloserine on cognition is required, particularly in populations for whom cognition is impaired.

### Limitations

The THINC-it has a number of strengths in its quick administration, repeatability,^15^ and clinical relevance,^14–16^ however it was designed as a screening tool and confirmation with more robust neuropsychological testing is warranted. In particular, we note the controversy within the literature in regards to the 1-Back as a measure of working memory and acknowledge that performance on the 1-Back can be interpreted as improvements in attention.^34^

Further, although we did not see evidence of an acute effect of D-cycloserine in healthy individuals relative to placebo, this may reflect a ceiling effect. We cannot exclude that subchronic D-cycloserine dosing may result in cognitive enhancement in the absence of iTBS. An additional limitation of these studies is the small sample size for both samples. Future studies to determine whether D-cycloserine adjunctive to iTBS improves cognition should consider enrichment for cognitive impairment, and power studies for small effect sizes consistent with literature on TMS and cognitive function.

## Conclusion

Succinctly, an intersectional strategy pairing noninvasive neurostimulation with NMDA receptor agonism may restore cognitive function in MDD. These findings highlight the potential of pharmacologically enhanced noninvasive neurostimulation to improve specific symptom domains in MDD, with potential relevance to other conditions associated with cognitive impairments.
